# Deflections and Stresses in Rectangular, Circular and Elliptical Insulating Glass Units

**DOI:** 10.3390/ma15072427

**Published:** 2022-03-25

**Authors:** Zbigniew Respondek, Marcin Kozłowski, Maciej Wiśniowski

**Affiliations:** 1 Faculty of Civil Engineering, Czestochowa University of Technology, Dąbrowskiego 69, 42-201 Częstochowa, Poland; 2 Department of Structural Engineering, Faculty of Civil Engineering, Silesian University of Technology, Akademicka 5, 44-100 Gliwice, Poland; marcin.kozlowski@polsl.pl; 3 Department of Mechanics and Bridges, Faculty of Civil Engineering, Silesian University of Technology, Akademicka 5, 44-100 Gliwice, Poland; maciej.wojciech.wisniowski@polsl.pl

**Keywords:** glass in building, insulated glass units, climatic loads, finite element

## Abstract

Insulating glass units (IGUs) are construction elements that react to climatic loads in a specific way. Under the influence of changes in atmospheric pressure and temperature, as well as the effect of wind, the gas closed in the tight gap between the glass panes changes its pressure, which affects the resulting static quantities of the loaded IGUs. The calculation models described in the literature mostly concern rectangular units, however, other shapes are being implemented more and more often in modern architecture. The aim of the article was to propose analytical and numerical models of circular and elliptical IGUs and to compare their results in terms of deflections and stresses with static values for square and rectangular units. Calculation examples were presented for various dimensions of IGUs loaded with changes in atmospheric pressure and an external wind effect. For elliptical IGUs, only the numerical calculations were presented, as it is not possible to formulate an applicable deflection function practically. The results were summarized in the form of tables and graphs, which illustrate the percentage differences between the deflection and stress values for the rectilinear and curvilinear shapes of IGUs for various dimensions and types of loads. It was found that in a single circular glass pane the maximum deflection is 4.2% greater, and the maximum stress is 13% greater than in a square unit of the same dimension. Meanwhile, in a circular, symmetrically loaded double-glazed IGU, the deflection in the circular IGU is smaller by 8–9% than in the square unit and the stress is practically identical.

## 1. Introduction

Insulating glass units (IGU) are classic constructions used to fill standard windows and glass facades. Their task is primarily to reduce heat loss in buildings. A unit consists of two or more glass panes sealed together at the periphery by an edge spacer. Thanks to this connection, the gap between the panes can be filled with gas that has better thermal insulation than air—argon is most often used. In addition, thanks to the tightness of the gap, delicate, thin low-emission or selective layers (coatings with a thickness of several dozen nanometers) can be placed onto the surfaces of the glass panes, which quickly corrode if exposed to atmospheric conditions. These layers have low infrared emissivity, which makes the escape of heat from the inside of the building difficult. In this way, the thermal transmittance of the glazing *U*_g_ significantly decreases. For example, a unit consisting of two 4 mm thick panes with a 16 mm gap filled with argon (IGU 4-16-4) without low-E coating shows *U*_g_ ≈ 2.6 W/(m^2^·K), while adding one low-E layer results in a *U*_g_ ≈ 1.1 W/(m^2^·K). Thus, heat losses are considerably limited (values calculated according to [1]).

It can, therefore, be concluded that IGUs were constructed to meet the need to increase the thermal insulation of transparent building envelopes, which were previously “energy-weak elements” of a building. This is especially important in countries with a cold climate, where the thermal protection of a building is of key importance.

However, it quickly turned out that the tightly-sealed, gas-filled gap is an element that influences the loads and static quantities (deflection and stress) in the glass panes. First of all, temporary changes in external conditions, regarding atmospheric pressure and temperature, resulting in a pressure difference between the gas closed in the gap and the surroundings. As a result, the panes of the unit bend, changing the gas pressure in the gap and its volume. This change in the gas pressure (interaction) results in a secondary load which partially reduces the external load. Such a system remains in a state of temporary equilibrium—smooth changes of external factors result in a smooth change of this state. In general, it can be seen that an increase in air temperature in the gap or a decrease of atmospheric pressure cause the component panes to take a convex form of deflection—the opposite action leads to a concave form of deflection in the IGU (see figures, for example in [2,3,4,5]). These loads are characteristic only for IGUs—if the gap was not airtight, the internal pressure would quickly equalize with the atmospheric pressure and these loads would not form. It can, therefore, be concluded that this is an unfavorable side effect of the tightly sealed nature of the gap. This effect can be visually manifested, for example, by distortion of the image reflected from the deflected glass panes.

The situation is different in the case of uniformly distributed loads (e.g., wind pressure)—here the tightly-sealed gap works favorably, the external load is distributed over all the component panes of the unit.

The theoretical solution to the problem comes down to the determination of the resultant load acting on each of the glass panes of the unit. There are several mathematical models in the literature—all of them assume that the gas in the gaps satisfies the ideal gas equation. So, for each gap:

(1) $$\frac{p_{0}\cdot v_{0}}{T_{0}}=\frac{p_{\mathrm{op}}\cdot v_{\mathrm{op}}}{T_{\mathrm{op}}}=\mathrm{const}$$

where:

*p*_0_, *T*_0_—initial parameters of gas in the gap: pressure [kPa] and temperature [K], parameters obtained in the production process, it is assumed that the panes delimiting the gap are perfectly parallel to each other,
*v*_0_—initial volume of the gap [m^3^], resulting from its nominal dimensions,
*p*_op_, *T*_op_, *v*_op_—operating parameters—similarly; they reflect the state of temporary equilibrium between the parameters of gas in the gap and the external loads.

The analytical model assuming the linear dependence of the glass panes deflection on the resultant load for double-glazed units was presented by Solvason in 1974 [2]. However, the best known is the model developed by Feldmaier, described in several of his works—a summary containing practical formulas and examples, also for triple-glazed units, is presented in the article [3]. Based on Feldmaier’s works, suitable formulas for practical calculations can be found in the guidebook [4]. The calculation procedures for multi-glazed units based on the classic model of a simply supported plate [5] are described in articles [6,7]. Galuppi and Royer-Carfagi developed a model of pressure changes in gaps of multi-glazed IGUs using Green’s functions [8]. In the article [9], Stratiy presented experimental research of the deflection of double-glazed units and compared the results with theoretical calculations according to various assumptions (simple or rigid support, small or large deflections). Bedon and Amadio [10], on the basis of numerical (FEM) and analytical analyzes, linked the problems of glass deflection under climatic load with the buckling phenomenon of IGUs. The FEM-based numerical calculations of IGUs are presented in [11,12].

Experimental research in this area was also conducted. Hart et. all [13] investigated the actual deflections in IGUs installed in various locations in the USA. Research on climatic loads, measuring the pressure in IGUs gaps located in climatic chambers, was presented in articles [14,15]. Bedon and Amadio presented experimental tests and modeling of edge connection efficiency for different types of spacers in IGUs [16,17]. Experimental studies and numerical analysis of IGUs bending with the use of cold-bent glass are presented in [18].

Until recently, the most frequently used construction in Central Europe was the double-glazed IGU 4-16-4. Currently, due to the continuous tightening of regulations on thermal protection of buildings, the 4-16-4-16-4 triple-glazed units with two low-emission layers (*U*_g_ = 0.6–0.7 W/(m^2^·K)) are mass-produced and installed. Quadruple-glazed units are used to a lesser extent; however, research is carried out with the use of 6-pane glazing [19]. Increasing the number of panes is beneficial in the context of IGUs thermal insulation. It increases the total thickness of the gas in the gaps, and thus the climatic loads of the panels in the sets increase.

In the above-mentioned analytical and numerical models, static quantities in rectangular IGUs are considered. However, other shapes of units are possible, in particular, circircular and elliptical. Such units are offered on the market by many manufacturers as custom-made orders.

The aim of the article is to propose analytical and numerical models of circular and elliptical IGUs. Then, on this basis the article is aimed at the comparison of the values of deflection and stress in IGUs loaded with static values for square and rectangular units. In addition, the analytical models are supplemented by taking into account the self-weight of component glass panes in the case of non-vertical positioning of IGUs. The work is part of an ongoing research project “Analysis of internal pressure in 3D bent insulated glass units” financed by The National Science Center (NCN) within the MINIATURA 5 program.

## 2. Methodology of Research

In the scope of the research task carried out in this work, a basic analytical model for calculating deflection and stress in rectangular IGUs with an unlimited number of gaps was adopted, taking into account the possibility of a non-vertical positioning of the unit. Next, an analytical model for determining static quantities in circular IGUs was developed. In parallel, a numerical model for determining static quantities in square and circular IGUs was developed and compatibility with the analytical model was verified.

For example, using IGUs with different dimensions, the static quantities (resultant load, deflection, stress) were compared in square and circular IGUs loaded with changes in atmospheric pressure and wind pressure.

Finally, a numerical model was used to identify and compare static quantities in rectangular and elliptical IGUs.

### 2.1. Analytical Model of a Rectangular Multi-Glazed IGU

**Figure 1:**
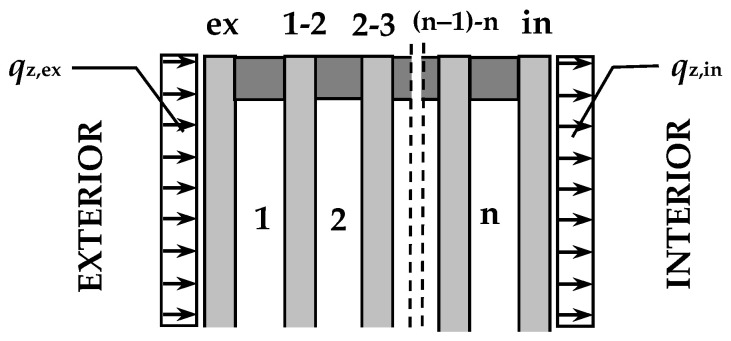
Index markings of gaps and glass panes in the unit.

The base model for determining static quantities in a rectangular IGU is described in [7,20]. The unit consists of *n* slots and (*n* + 1) glass plates; the subscript symbols for the gaps and plates are described in Figure 1.

It was assumed that the initial parameters of gas *p*_0_, *T*_0_ are known and are the same for all gaps; with these parameters and the vertical positioning of the IGU, the component plates are undeformed and parallel to each other. Under operating conditions, the glass panes may be subject to temporary changes in atmospheric pressure, changes in gas temperature in the gaps, as well as surface loads per area (mainly wind) marked as *q*_z,ex_, *q*_z,in_ [kN/m^2^] in Figure 1. It was also assumed that the deflections of the panes in the unit are small, i.e., the relationship between the deflection and the resultant load of individual component panes is linear; according to [21] the linear approximation is sufficient if the deflection does not exceed the plate thickness.

The convention is that loads and deflections are positive when facing the interior (left to right as in Figure 1, or top down for horizontally placed panes).

The model was experimentally validated for double-glazed IGUs [22]. In the tests, the asymmetric surface load was simulated with a water column 0–35 cm high (the IGU formed the bottom of the water tank). Based on the measured deflections, the percentage value of the load transferred to the gas-coupled plate (not directly loaded) was estimated. The experimental values differed from the theoretical maximum by 7.44%, and on average by 3.15%.

**Figure 2:**
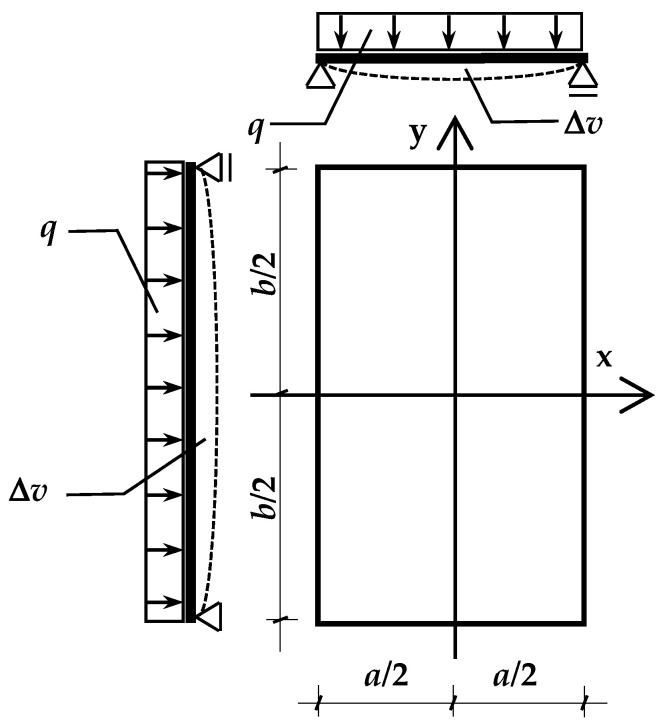
Adopted coordinate system and method of supporting the rectangular IGU.

In operating conditions, each of the panes in the unit bends under the action of the resultant load *q* [kN/m^2^] acting on the surface. This causes a change in the volume of the gaps adjacent to the given pane. Assuming the coordinate system as shown in Figure 2, the change in the gap volume Δ*v* [m^3^] caused by the deflection of one of the limiting glass plates is expressed by:

(2) $$\Delta v=\pm \int_{\frac{-b}{2}}^{\frac{b}{2}}\int_{\frac{-a}{2}}^{\frac{a}{2}}w\left(x,y\right)\mathrm{dxdy}=\pm \alpha_{v}\cdot q$$

where:

*a*, *b*—width and length of glass panes [m],
*w(x,y)*—function of deflection [m],
*α*_v_—proportionality factor [m^5^/kN],

In the analysis, it was assumed that the change in volume Δ*v* is positive if it causes an increase in the volume of the adjacent gap.

The methodology of calculating Δ*v* is described in more detail in [20]. After applying the function of deflection from Timoshenko and Woinowsky-Krieger [5] and the appropriate transformations, the following was obtained:

(3) $$\alpha_{v}={\alpha^{\prime }}_{v}\cdot \frac{a^{6}}{D}\;\mathrm{with}\;D=\frac{E\cdot d^{3}}{12\cdot \left(1-\mu^{2}\right)}$$

where:

*α*′_v_—dimensionless coefficient [-], see Table 1,

*D*—flexural rigidity of glass pane [kNm],

*d*—glass pane thickness [m],

*E*—Young’s modulus of glass [kPa],

*μ*—Poisson’s ratio [-].

Formula (1) gives the equation of state which for each gap in the set can be formulated as:

(4) $$p_{0}\cdot v_{0}\cdot T_{\mathrm{op}}=p_{\mathrm{op}}\cdot \left(v_{0}+\sum \Delta v\right)\cdot T_{0}$$

where:

∑Δ*v*—change in gap volume caused by deflection of both panes limiting the gap [m^3^].

The equations of state for all the gaps in the unit form a system of n quadratic equations, the solution of which allows the operating pressure in each IGU gap to be determined. The system of equations for IGUs located vertically is presented in [7].

Horizontal and diagonal glazing is more and more often used in practice—for example, winter garden coverings, glazed patios, or glass roofs over utility rooms. In the case of double-glazed IGUs, taking into account the self-weight of glass plates is also not a problem—the perpendicular component *q*_γ_ [kN/m^2^] can be included in the loads *q*_z,ex_ and *q*_z,in_. For each of the plates, the value of *q*_γ_ is:

(5) $$q_{\gamma }=\gamma \cdot d\cdot \cos\varphi$$

where:

*γ*—the specific gravity of the glass [kN/m^3^],

*φ*—the angle of slope of the IGU to the horizontal [deg].

It is different in the case of multi-glazed IGUs—the self-weight should be included in the system of equations. Then it can be written as follows:

(6a) $$p_{1}\cdot \left[v_{01}+A_{1}\cdot \alpha_{v,\mathrm{ex}}+B_{1}\cdot \alpha_{v,1-2}\right]-\frac{p_{0}\cdot v_{01}\cdot T_{1}}{T_{0}}=0$$

(6b) $$\;\;\;p_{2}\cdot \left[v_{02}+A_{2}\cdot \alpha_{v,1-2}+B_{2}\cdot \alpha_{v,2-3}\right]-\frac{p_{0}\cdot v_{02}\cdot T_{2}}{T_{0}}=0$$

(6c) $$\;\;\;\;\;\;\;\;p_{n}\cdot \left[v_{0n}+A_{n}\cdot \alpha_{v,\left(n-1\right)-n}+B_{n}\cdot \alpha_{v,\mathrm{in}}\right]-\frac{p_{0}\cdot v_{0n}\cdot T_{n}}{T_{0}}=0$$

where:

*p*_1_, …, *p_n_*—unknown operating pressure of gas in the gaps [kPa],

*p*_a_—current atmospheric pressure [kPa],

*T*_1_, …, *T_n_*—operating gas temperature in the gaps [K],

*v*_01_, …, *v*_0*n*_—the gap volume [m^3^],

*α*_v,ex_, *α*_v,1−2_, …, *α*_v,(*n*__−1)−*n*_, *α*_v,in_—proportionality factors for individual components of the unit, [m^5^/kN].

In the above formulas there are auxiliary parameters:

(7a) $$A_{1}=p_{1}-c_{ex}\;\;\;\;\;\;\;\;\;\;B_{1}=p_{1}-p_{2}+q_{\gamma ,1-2}$$

(7b) $$A_{2}=p_{2}-p_{1}-q_{\gamma ,1-2}\;\;\;\;\;\;\;\;B_{2}=p_{2}-p_{3}+q_{\gamma ,2-3}$$

(7c) $$A_{n}=p_{n}-p_{\left(n-1\right)}-q_{\gamma ,\left(n-1\right)-n}\;\;\;\;\;\;\;\;\;B_{n}=p_{n}-c_{in}$$

With

(7d) $$c_{\mathrm{ex}}=p_{a}+q_{z,\mathrm{ex}}+q_{\gamma ,\mathrm{ex}}\;\;\;\;\;\;\;\;c_{\mathrm{in}}=p_{a}-q_{z,\mathrm{in}}-q_{\gamma ,\mathrm{in}}$$

The system of equations for *n* > 1 has no analytical solutions—the system should be solved numerically by iteration. For *n* = 1 (double-glazed IGU) there is an analytical solution:

(8) $$p_{1}=\frac{A}{2\cdot B}+\sqrt{{\left(\frac{A}{2\cdot B}\right)}^{2}+\frac{p_{0}\cdot v_{01}\cdot T_{1}}{B\cdot T_{0}}}$$

With

(9) $$A=c_{\mathrm{ex}}\cdot \alpha_{v,\mathrm{ex}}+c_{\mathrm{in}}\cdot \alpha_{v,\mathrm{in}}-v_{01}\;\;\;B=\alpha_{v,\mathrm{ex}}+\alpha_{v,\mathrm{in}}$$

The knowledge of the operating pressure in the gaps allows the resultant load *q* for each of the component panes to be determined:

(10) $$q_{\mathrm{ex}}=c_{\mathrm{ex}}-p_{1}\;q_{1-2}=p_{1}-p_{2}\;\ldots ,\;q_{\mathrm{in}}=p_{n}-c_{\mathrm{in}}$$

Then, the static quantities in the individual panes of the unit based on generally known equations of plate theory can be estimated. Deflection *w* [m] at the center of the glass pane is:

(11) $$w={\alpha^{\prime }}_{w}\cdot \frac{q\cdot a^{4}}{D}$$

where:

*α*′_w_—dimensionless coefficient [-], see Table 1.

Stress *σ* [m] in the center of the pane in the direction of the x and y axes:

(12) $$\sigma_{x}=k_{x}\cdot \frac{q\cdot a^{2}}{d^{2}}\;\;\;\;\;\;\;\;\;\;\;\;\sigma_{y}=k_{y}\cdot \frac{q\cdot a^{2}}{d^{2}}$$

where:

*k*_x_, *k*_y_—dimensionless coefficients [-], for *μ* = 0.2 see Table 1.

The presented model allows the estimation of the deflection and stress in IGUs of various structures subjected to loads related to the temporary changes of atmospheric pressure and temperature, as well as the effects of wind.

### 2.2. Analytical Model of a Circular Multi-Glazed IGU

**Figure 3:**
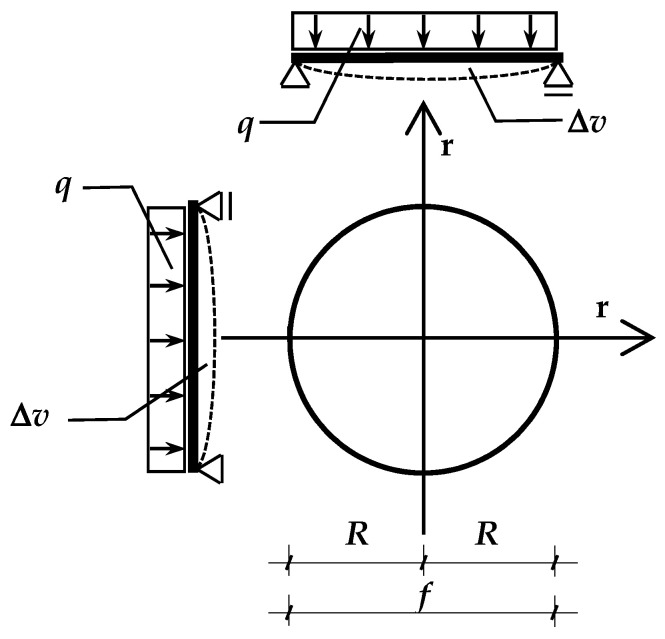
Adopted coordinate system and method of supporting the circular IGU.

To create an analogous calculation model for IGUs with a shape other than rectangular, it is necessary to formulate the function of deflection in an analytical form and integrate it over the surface of the pane. This is possible with the circular shape of the IGU. In this case, it is convenient to consider a single glass pane in the radial system with the coordinate r (Figure 3). The pane has the radius *R* [m] and the diameter *f* = 2*R*.

Timoshenko and Woinowsky-Krieger [5] give the deflection function in the form:

(13) $$w\left(r\right)=\frac{q}{64\cdot D}\cdot \left(R^{2}-r^{2}\right)\cdot \left(Z\cdot R^{2}-r^{2}\right)$$

With

(14) $$Z=\frac{5+\mu }{1+\mu }$$

Then, the maximum deflection *w*_r_ [m] and the stress *σ*_r_ [kPa] in the center of the pane can be formulated:

(15) $$w_{r}=\frac{Z}{64}\cdot \frac{q\cdot R^{4}}{D}=\frac{Z}{1024}\cdot \frac{q\cdot f^{4}}{D}={\alpha^{\prime }}_{w,r}\cdot \frac{q\cdot f^{4}}{D}$$

(16) $$\sigma_{r}=\frac{3\cdot \left(3+\mu \right)}{8}\cdot \frac{q\cdot R^{2}}{d^{2}}=\frac{3\cdot \left(3+\mu \right)}{32}\cdot \frac{q\cdot f^{2}}{d^{2}}=k_{r}\cdot \frac{q\cdot f^{2}}{d^{2}}\;$$

where:

*α*_w,r_, *k*_r_—dimensionless coefficients [-].

For *μ* = 0.2, the values of the coefficients are as follows: *α*′_w,r_ = 0.004231; *k*_r_ = 0.3. A comparison with the data from Table 1 shows that for the same surface load *q*, the maximum deflection in a circular pane with a diameter *f* is 4.2% greater, and the maximum stress is 13% greater than in a square plate with a side dimension *a* = *f*.

Of course, this fact does not translate directly into static quantities in IGUs. The greater deflection vulnerability results in increased gas interaction in the gaps, whereby the resultant loads for the panes are different for the same external load.

It should be noted that less rigid and larger panes are more susceptible to deflection—the problem is, therefore, complex and requires analysis each time the size, shape and structure of the IGU is changed.

In order to determine the proportionality coefficient *α*_v,r_ for the change in the gap volume caused by the deflection of the loaded circular glass, the function of deflection should be integrated.

(17) $$\Delta v=\int_{0}^{R}w\left(r\right)\cdot 2\pi r\;\mathrm{dr}=\alpha_{v,r}\cdot q$$

After the appropriate transformations:

(18) $$\Delta v=\frac{\pi }{2048}\cdot \left(\frac{Z}{2}-\frac{Z+1}{4}+\frac{1}{6}\right)\cdot \frac{q\cdot f^{6}}{D}={\alpha^{\prime }}_{v,r}\cdot \frac{q\cdot f^{6}}{D}$$

where:

*α*′_v,r_—dimensionless coefficient [-]; for *μ* = 0.2 → *α*′_v,r_ = 0.001534.

The further procedure is the same as in the case of a rectangular glass pane. In order to determine the operating gas pressure in each gap and the resultant load for each component pane, formulas 6 ÷ 10 can be used, however, for each pane, instead of the *α*_v_ factor, *α*_v,r_ should be calculated from the formula:

(19) $$\alpha_{v,r}={\alpha^{\prime }}_{v,r}\cdot \frac{f^{6}}{D}$$

Maximum deflection and stress should be calculated from Formulas (15) and (16).

### 2.3. Numerical Model for Determining Static Quantities in IGUs

**Figure 4:**
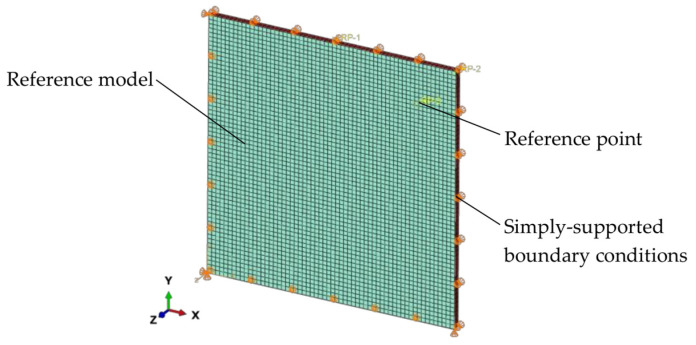
A 3D reference model. Square, double-glazed IGU 4-16-4 with dimensions 0.4–1.2 m. Material data and boundary conditions in the text.

A reference (rectangular) finite model (FE) was developed and is shown in Figure 4. It is composed of two shells representing the glass panes and a set of shells (representing a gasket) that closes the unit around its periphery. The glass panes and the spacer were modeled using shell elements with reduced integration (S4R element type from Abaqus library [23]). This was performed for a practical reason—to include the possible transmission of loads from one glass pane to the other only via the gap. In the reference model, all edges of the glass panes were simply-supported. The same assumptions were used for the circular and elliptical models. In the analyses, large deflection theory was adapted.

Glass was represented using linear elastic material properties with the density ρ = 2500 kg/m^3^, the nominal Young’s modulus *E* = 70 GPa and Poisson’s ratio *μ* = 0.20 [24]. For the spacer, equivalent linear-elastic materials were considered with *E* = 3.0 MPa and *μ* = 0.30 [25].

Special attention was paid to the description of load share effects as a result of a sealed, air-filled gap between the panes. A fluid cavity interaction (pneumatic gas law) was applied in the models [23], the approach provides the coupling between the deformation of the fluid-filled assembly and the pressure exerted by the contained fluid on the gap boundary of the structure [26]. The boundary of the fluid cavity was defined by element-based surfaces (the glass panes and spacer) with normal directions pointing to the inside of the gap. This approach requires a definition of a single node (cavity reference node), which is associated with the fluid cavity. The reference node has a single degree of freedom representing the pressure inside the fluid cavity. In the FE simulations for a fluid cavity, calculations are performed using volume elements (internally created by Abaqus) using the surface facet geometry and the cavity reference node. In the simulations, the following physical constants were taken into account: the universal gas constant Ru = 8.314 J/(K·mol) [27] and the molecular weight of dry air M_air_ = 28.97 g/mol [28]. It should be noted that the type of gas does not affect the calculation results, as the pressure is the parameter that matters. However, declaring this data is necessary for the correct operation of the software.

**Figure 5:**
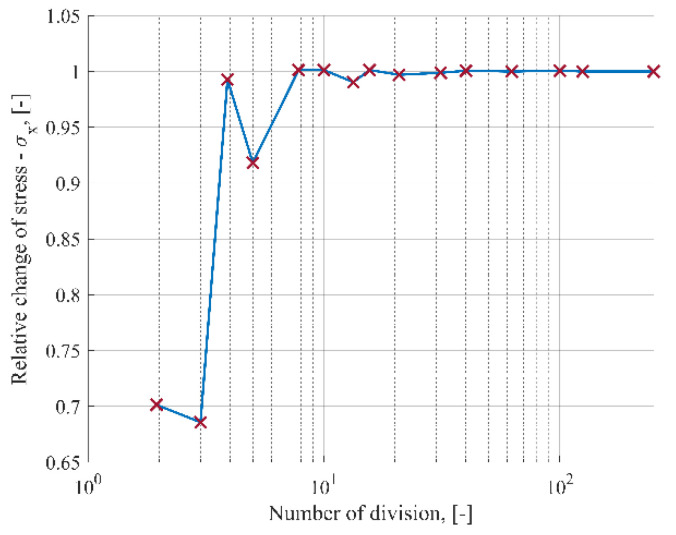
Results of the mesh convergence study.

Following a mesh convergence study, which was aimed at the verification of the mesh quality, a regular mesh pattern was applied to the glass panes and the spacer. Figure 5 shows the relationship of the relative change of the normalized maximum principal stress in glass to the number of divisions of the edge along the periphery of the glass panes. From the mesh convergence study, it was found that the model with 100 finite elements along the edge of the pane converges to a sufficient degree. The same mesh was used for the circular and elliptical models.

## 3. Results

### 3.1. Comparison of Static Quantities in Square and Circular IGUs

On the basis of the previously presented models, calculations were made for exemplary double-glazed IGUs. The current most commonly produced unit was adopted: thickness of component panes *d* = 4 mm, gap thickness *s* = 16 mm.

Simple support boundary conditions at the edge of IGUs were assumed as well as the initial conditions (i.e., the state without deformations and stresses): *p*_0_ = *p*_a_ = 100 kPa, *T*_0_ = 20 °C = 293.15 K. Glass parameters: *E* = 70 GPa, *μ* = 0.2.

**Figure 6:**
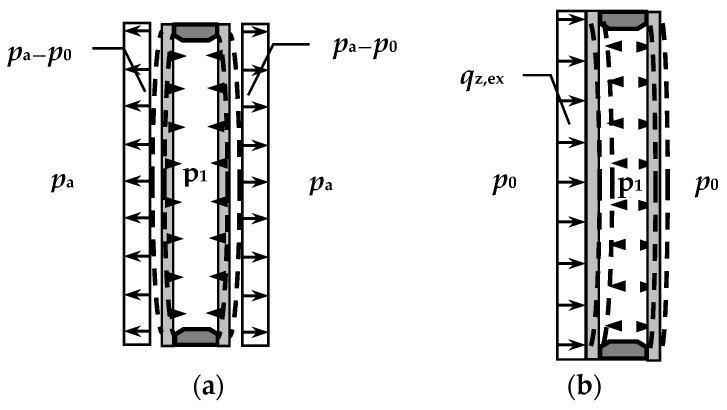
Climatic loads acting on IGUs: (**a**) load with a decrease in external atmospheric pressure; (**b**) wind pressure load.

Two typical climatic loads were considered (Figure 6):

- symmetrical load—a decrease in the external atmospheric pressure by 5 kPa, i.e., *p*_a_ = 95 kPa (similar effects occur with an increase in gas temperature in the gap, e.g., by 15.5 K with dimensions of 0.8 × 0.8 m), in the case of a double-glazed IGU, the deflection and the stress are the same (in absolute value) in both component panes;
- wind pressure load—assumed at *q*_z,ex_ = 0.3 kN/m^2^, which corresponds to wind speed of about 80 km/h [29]; due to the interaction of the gas in the gap, the component glass panes are coupled together; with large IGUs, the external load is distributed almost equally on both panes, with smaller sizes, most of the load is taken by the directly loaded pane; the results presented below are for the more loaded pane.

**Figure 7:**
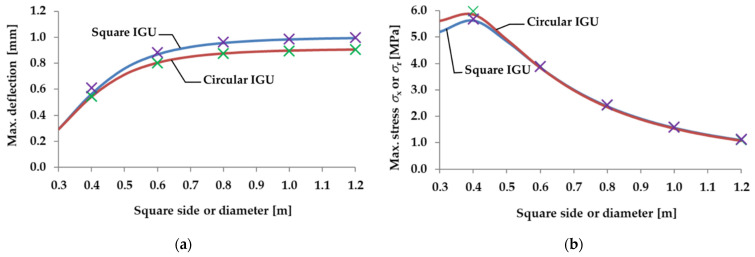
Static quantities in IGUs loaded with a 5 kPa atmospheric pressure decrease for various dimensions of square and circular units: (**a**) maximum deflection; (**b**) maximum stress. The solid line marks the results of analytical calculations, the crosses (purple for square IGUs, green for circular IGUs)—the results of the numerical model. A detailed description of the geometric and material assumptions of the model is included in the text.

**Figure 8:**
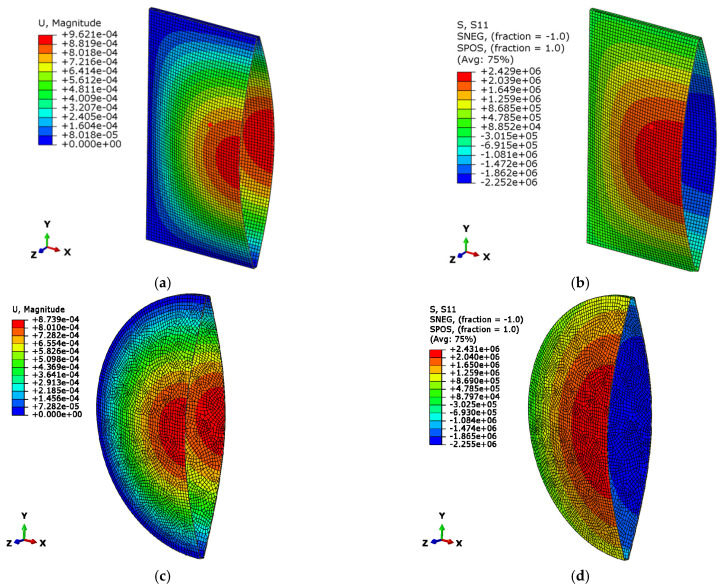
Examples of the numerical analysis results for IGUs with dimension 80 cm: (**a**) deflection map for a square IGU, (**b**) stress map *σ*_x_ for a square IGU, (**c**) deformation map for a circular IGU, (**d**) stress map *σ*_r_ for a circular IGU.

Figure 7 shows the analytical (lines) and numerical (cross markers) calculations of the maximum deflection and stress in the IGUs loaded with a decrease in atmospheric pressure. Figure 8 shows an example of the results of the numerical calculations for selected glass panes: rectangular and circular, with a side length or diameter of 80 cm. The drawings show maps of displacements and stresses *σ*_x_ or *σ*_r_ for a load with a 5 kPa decrease in the external atmospheric pressure.

Based on the obtained results, it was found that, as shown in Section 2.2, a single circular pane is more susceptible to deflection, which results in an increased interaction of gas in the gap—consequently, for larger dimensions, the deflection in the circular IGU is 8–9% smaller than in the square IGU and the maximum stress diagrams practically coincide.

The influence of IGU dimensions on static quantities is characteristic for a symmetrical type of load: with an increase in dimensions, the interaction increases significantly—deflections hardly increase and stresses decrease; for each IGU there is a critical dimension for which the stress is greatest; the calculations show that with dimensions close to the critical, the stresses in the circular IGU are about 5% higher than in the square units.

The models used are almost compatible for most of the data; for deflection, this is only the case with the 0.4 m dimension units, the results from the numerical calculations are 7.5% higher than those calculated analytically; for stresses, the numerical results are greater than the analytical ones by 1–3%.

**Figure 9:**
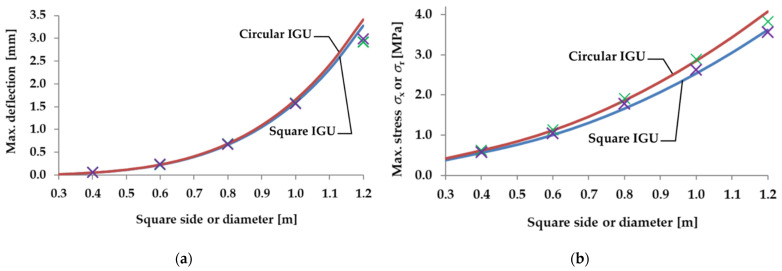
Static quantities in IGUs loaded with a wind pressure of 0.3 kN/m^2^ for various dimensions of square and circular panes: (**a**) maximum deflection; (**b**) maximum stress. Lines and cross markers—as in Figure 7. A detailed description of the geometric and material assumptions of the model is included in the text.

Figure 9 shows the static quantities in IGUs loaded with wind pressure. Based on the obtained results, it was found that as previously mentioned, for larger dimensions, the external load is distributed equally on both component panes—in the range of small deflections, the static quantities increase exponentially.

In the case of an asymmetric load, the increased interaction in the circular IGUs works differently than in the previous case—here the deflection graphics almost coincide, while the stress, for larger dimensions, is approx. 13% higher in the circular unit (i.e., as in the case of a single pane, see Section 2.2).

The dimension of 1.2 m shows the difference between the analytical and numerical results; the numerically calculated deflection is clearly smaller; the non-linear behavior of the panes is shown here (second-order effects related to deflections of the panes close to half of their thickness, which determines the limits of applicability of the analytical model).

In the range 0.4–1.0 m the numerical results do not differ by more than 4% from the analytical results; the exceptions are the stresses in square IGUs with the 0.8 m dimension, the numerical values of which are 7.3% greater than the analytical values.

### 3.2. Comparison of Static Quantities in Multi-Glazed IGUs

The comparison of static values in IGUs with a different number of gaps was carried out using the analytical method. Table 2 and Table 3 present the appropriate summary of the results for the three selected dimensions for both the loads considered and the percentage change of these values in the circular IGU in relation to the square unit. The presented results refer to the most loaded pane.

Based on the data obtained, it was found that in the case of symmetrical loading, an increase in the number of gaps, and thus, the total thickness of the gas layers, increases the maximum resultant load, deflection and stress. For larger dimensions, the static quantities increase approximately linearly. In the case of an asymmetric load, an increase in the number of gaps, and thus glass panes, almost always results in a reduction of the static values. This is especially evident with larger dimensions, where the external load is distributed almost equally over all panes;

The results confirmed the supposition that the greater susceptibility of the circular IGUs to deflection resulted in increased gas interaction, i.e., a lower resultant load than in square IGUs; this may translate into the resultant deflection and stress in various ways, as shown by the presented data.

### 3.3. Comparison of Static Quantities in Rectangular and Elliptical IGUs

The general analytical solution for rectangular IGUs is presented in Section 2.1. Table 4 shows a comparison of the analytical and numerical results for the exemplary IGUs loaded as before, for the ratio of dimensions *b*/*a* = 1.5 and *b*/*a* = 2. The data shows that the values of deflection or stress estimated by both methods differ by a maximum of 3.2%, which proves the good compatibility of these methods.

The issue of determining the deflection function for loaded elliptical plates was analyzed by Sato in the article [30]. According to the provided solution, the deflection function depends on the position of a given cross-section in the elliptical coordinate system. It has such a complex form that it is not possible to analytically derive the integral needed to determine the volume change of the loaded plate. For this reason, the comparison of the sizes of static rectangular and elliptical plates was performed only numerically. The results of the calculations are presented in Table 5. In the case of an ellipse, the symbols *a* and *b* denote the minor and the major axis, respectively.

Based on the data obtained, it was found that in the case of a symmetrical load, a similar effect as for square and circular panes was obtained: the deflections in the elliptical IGU are smaller than in the rectangular units, the maximum stresses in both types do not differ much.

In the case of an asymmetric load, the findings were different, the deflections and stresses in the elliptical IGU are smaller than in the rectangular unit; it can generally be said that increasing the b/a dimension ratio leads to an increase (in absolute value) of the percentage change in the static quantities in the rectangle-ellipse comparison.

## 4. Discussion

Estimating static quantities in insulating glass units is a complex problem. The resulting values of deflection and stress depend on the degree of interaction of the gas gap with external loads. Both the dimensions of the IGU and its structure have an influence on the results. In principle, each case should be considered individually. While the examples shown cover only the two basic types of loads, the possibilities of the proposed models are much greater. They can also be used to estimate the static values in IGUs loaded with gas temperature changes in the gaps, it is also possible to consider any combination of climatic loads acting simultaneously, which is important because the problem is non-linear and the application of the superposition principle does not always give a sufficient approximation. With the methods, it is also possible to analyze IGUs with component glass panes of different stiffness.

The two methods of calculating static values in the insulating glass units presented in the article show advantages and disadvantages. The analytical method can be considered mathematically accurate, but it is limited to some cases. For example, in the case of large deflections or elliptical IGU shapes, it is necessary to use numerical methods based on FEM. Numerical methods also allow for a better presentation of the results in the context of the graphical presentation of the analyzed quantities in the entire element. However, the accuracy of numerical methods depends on many factors, such as the correct selection of the mesh size and the correct definition of the calculation assumptions regarding the boundary conditions and materials’ parameters.

The Abaqus environment offers much greater possibilities compared to the MES engineering programs dedicated to the calculations of insulating glass units (e.g., SJ-Mepla) available on the market. Their biggest disadvantage is the inability to take into account the rheological effects of materials, e.g., silicone sealing, and the inability to conduct coupled mechanical-thermal analyses.

The authors see the need to continue work on this topic, in particular, the implementation of multi-glazed IGUs in the Abaqus software, IGUs with curved glass panes and implementing important factors in the calculations, such as viscoelastic properties of the interlayer in the case of laminated glass and the possibility of support boundary conditions other than simple support boundary conditions at the edges, in particular, the assumption of flexible supports. Experimental research in this area is also planned.

## 5. Conclusions

The article proposes both analytical and numerical calculation models for determining static quantities in climatically loaded square, rectangular and circular IGUs. Due to the limited possibilities of the analytical solutions, elliptical IGUs were solved only numerically. The presented examples show the influence of gas interactions in IGU chambers of various shapes on two common types of climatic loads. The gas interaction affects the value of the resultant load on individual components of the set. The static values in IGUs differ from those analyzed for individual panes, which has been shown in the article in the form of graphs and tables.

For example, a single circular plate is more susceptible to deflection than a corresponding square plate, it also shows 13% higher stress in glass. While in the case of a symmetrical load on the IGU, the deflections are greater in the square unit, and the stresses in both structures are almost the same. In elliptical IGUs, the static values are smaller than in the corresponding rectangular units, which is especially evident in the large *b*/*a* dimension ratio.

The calculations according to the analytical model can be treated as more accurate in mathematical terms, but their scope is limited. In terms of the subject of this analysis, the main limitation is the assumption of a linear deflection relationship. In the context of the examples presented in the article, it can be concluded that the analytical methods neglecting large deformations are sufficiently precise in the range up to approx. 3 mm, i.e., ¾ of the pane thickness.

Numerical methods allow the analysis of a wider range of assumptions, such as elliptical IGUs, but their mathematical accuracy is lower and there is a greater possibility of making errors in defining the assumptions. Therefore, it is important that the numerical calculations are, to the greatest possible extent, validated with analytical methods, as carried out in this paper.

## Figures and Tables

**Table 1:** Dimensionless coefficients for the determination of static quantities in the IGUs.

b/a	1.0	1.1	1.2	1.3	1.4	1.5
***α*′_v_**	0.001703	0.002246	0.002848	0.003499	0.004189	0.004912
***α*′_w_**	0.004062	0.004869	0.005651	0.006392	0.007085	0.007724
***k*_x_**	0.265583	0.311731	0.355835	0.397214	0.435492	0.470517
***k*_y_**	0.265583	0.269151	0.269136	0.266305	0.261524	0.255476
**b/a**	**1.6**	**1.7**	**1.8**	**1.9**	**2.0**	**3.0**
***α*′_v_**	0.005659	0.006427	0.00721	0.008004	0.008808	0.017055
***α*′_w_**	0.008308	0.008838	0.009316	0.009745	0.010129	0.012233
***k*_x_**	0.502299	0.530948	0.556641	0.579589	0.600017	0.710253
***k*_y_**	0.248681	0.241524	0.234287	0.227166	0.220299	0.173499

**Table 2:** Comparison of static quantities in IGUs loaded with a decrease in atmospheric pressure from *p*_0_ = 100 kPa to *p*_a_ = 95 kPa.

Type of IGU	Square IGU			Circular IGU			Percentage Difference		
	*q* [kN/m^2^]	*w* [mm]	*σ*_x_ [MPa]	*q* [kN/m^2^]	*w* [mm]	σ_r_ [MPa]	for *q*	for *w*	for *σ*
Square side or diameter 0.4 m									
2-glazed	2.118	0.566	5.625	1.955	0.544	5.864	−7.73%	−3.88%	4.23%
3-glazed	2.965	0.793	7.874	2.800	0.780	8.400	−5.57%	−1.62%	6.67%
4-glazed	3.259	0.871	8.654	3.116	0.868	9.349	−4.36%	−0.37%	8.03%
Square side or diameter 0.7 m									
2-glazed	0.370	0.927	3.006	0.325	0.850	2.989	−11.96%	−8.29%	−0.55%
3-glazed	0.686	1.720	5.579	0.609	1.592	5.598	−11.17%	−7.46%	0.34%
4-glazed	0.941	2.359	7.652	0.843	2.203	7.748	−10.36%	−6.61%	1.26%
Square side or diameter 1.0 m									
2-glazed	0.094	0.985	1.565	0.082	0.896	1.545	−12.61%	−8.97%	−1.28%
3-glazed	0.128	1.951	2.562	0.112	1.779	2.533	−12.46%	−8.80%	−1.11%
4-glazed	0.270	2.820	4.482	0.237	2.581	4.447	−12.16%	−8.49%	−0.78%

**Table 3:** Comparison of static quantities in IGUs loaded with wind pressure *q*_z_ = 0.3 kN/m^2^.

Type of IGU	Square IGU			Circular IGU			Percentage Difference		
	*q* [kN/m^2^]	*w* [mm]	*σ*_x_ [MPa]	*q* [kN/m^2^]	*w* [mm]	σ_r_ [MPa]	for *q*	for *w*	for *σ*
Square side or diameter 0.4 m									
2-glazed	0.212	0.057	0.564	0.208	0.058	0.623	−2.33%	1.75%	10.33%
3-glazed	0.217	0.058	0.576	0.200	0.056	0.600	−7.79%	−3.93%	4.16%
4-glazed	0.203	0.054	0.539	0.198	0.055	0.595	−2.26%	1.82%	10.40%
Square side or diameter 0.7 m									
2-glazed	0.161	0.403	1.306	0.159	0.416	1.464	−0.79%	3.34%	12.06%
3-glazed	0.122	0.307	0.995	0.120	0.313	1.101	−2.06%	2.04%	10.64%
4-glazed	0.108	0.270	0.876	0.104	0.273	0.959	−3.12%	0.92%	9.43%
Square side or diameter 1.0 m									
2-glazed	0.153	1.595	2.534	0.152	1.658	2.857	−0.22%	3.94%	12.71%
3-glazed	0.106	1.107	1.759	0.105	1.146	1.974	−0.66%	3.50%	12.22%
4-glazed	0.084	0.882	1.402	0.083	0.908	1.564	−1.25%	2.88%	11.55%

**Table 4:** Comparison of the analytically and numerically calculated static quantities for rectangular IGUs.

Size a × b [m]	Analytical			Numerical			Percentage Difference		
	*q* [kN/m^2^]	*w* [mm]	*σ*_x_ [MPa]	*q* [kN/m^2^]	*w* [mm]	σ_r_ [MPa]	for *q*	for *w*	for *σ*
Decrease in atmospheric pressure from *p*_0_ = 100 kPa to *p*_a_ = 95 kPa									
0.4 × 0.6	1.390	0.707	6.540	1.321	0.729	6.469	−4.96%	3.15%	−1.09%
0.5 × 0.75	0.685	0.851	5.039	0.663	0.861	5.006	−3.32%	1.18%	−0.66%
0.6 × 0.9	0.357	0.919	3.779	0.349	0.924	3.771	−2.23%	0.56%	−0.21%
0.4 × 0.8	1.115	0.744	6.692	1.070	0.755	6.542	−4.06%	1.53%	−2.24%
0.5 × 1.0	0.529	0.861	4.959	0.515	0.865	4.884	−2.65%	0.47%	−1.52%
0.6 × 1.2	0.271	0.913	3.653	0.266	0.915	3.614	−1.69%	0.18%	−1.07%
Wind pressure *q*_z_ = 0.3 kN/m^2^									
0.4 × 0.6	0.191	0.097	0.897	0.196	0.100	0.925	2.85%	3.20%	3.16%
0.5 × 0.75	0.170	0.211	1.248	0.174	0.217	1.268	2.56%	2.87%	1.59%
0.6 × 0.9	0.160	0.412	1.696	0.163	0.421	1.740	1.73%	2.07%	2.57%
0.4 × 0.8	0.182	0.122	1.094	0.187	0.125	1.124	2.52%	2.78%	2.70%
0.5 × 1.0	0.165	0.269	1.549	0.169	0.275	1.584	2.08%	2.25%	2.26%
0.6 × 1.2	0.158	0.532	2.130	0.160	0.541	2.165	1.43%	1.60%	1.66%

**Table 5:** Comparison of numerically calculated static values for rectangular and elliptical IGUs.

Size a × b [m]	Rectangular IGU			Elliptical IGU			Percentage Difference		
	*q* [kN/m^2^]	*w* [mm]	*σ*_x_ [MPa]	*q* [kN/m^2^]	*w* [mm]	σ_r_ [MPa]	for *q*	for *w*	for *σ*
Decrease in atmospheric pressure from *p*_0_ = 100 kPa to *p*_a_ = 95 kPa									
0.4 × 0.6	1.321	0.729	6.469	1.350	0.662	6.496	2.21%	−9.17%	0.42%
0.5 × 0.75	0.663	0.861	5.006	0.662	0.792	4.983	−0.05%	−7.99%	−0.46%
0.6 × 0.9	0.349	0.924	3.771	0.344	0.853	3.726	−1.45%	−7.71%	−1.19%
0.4 × 0.8	1.070	0.755	6.542	1.179	0.707	6.631	10.15%	−6.38%	1.36%
0.5 × 1.0	0.515	0.865	4.884	0.564	0.826	4.959	9.54%	−4.59%	1.54%
0.6 × 1.2	0.266	0.915	3.614	0.290	0.879	3.665	8.99%	−3.95%	1.41%
Wind pressure *q*_z_ = 0.3 kN/m^2^									
0.40 × 0.60	0.196	0.100	0.925	0.189	0.0932	0.905	−3.40%	−6.89%	−2.14%
0.50 × 0.75	0.174	0.217	1.268	0.169	0.203	1.267	−2.92%	−6.23%	−0.08%
0.60 × 0.90	0.163	0.421	1.740	0.160	0.398	1.730	−2.18%	−5.55%	−0.57%
0.40 × 0.80	0.187	0.125	1.124	0.184	0.111	1.033	−1.50%	−11.44%	−8.10%
0.50 × 1.00	0.169	0.275	1.584	0.166	0.244	1.459	−1.45%	−11.35%	−7.89%
0.60 × 1.20	0.160	0.541	2.165	0.158	0.431	1.793	−1.15%	−20.37%	−17.18%

## Data Availability

Data will be provided on request.

## Nomenclature

*A*	auxiliary parameters [m^3^] or [kPa]
*a*	width or minor axis of ellipse [m]
*B*	auxiliary parameters [m^5^/kN] or [kPa]
*b*	length or major axis of ellipse [m]
*c*	auxiliary parameters [kPa]
*D*	flexural rigidity (of glass pane) [kNm]
*d*	thickness (of glass pane) [m] or [mm]
*E*	Young’s modulus [kPa] or [GPa]
*f*	diameter of circular IGU [m]
*i*	consecutive natural number
*k*	dimensionless coefficient regarding stress [-]
M	molecular weight [g/mol]
*p*	pressure [kPa]
*q*	resultant load (of glass pane) or outside load [kN/m^2^]
*R*	radius of circular IGU [m]
Ru	universal gas constant [J/(K·mol)]
*r*	coordinate in the polar system
*T*	temperature (of gas in the gap) [K] or [°C]
*U*	thermal transmittance [W/(m^2^·K)]
*v*	volume (of the gap) [m^3^]
*w*	deflection [m] or [mm]
*w*(*r*)	function of deflection (circular IGU), [m]
*w*(*x*,*y*)	function of deflection [m]
*x*-*y*	coordinate system
Greek Letters	
*α*	proportionality factor, [m^5^/kN]
*α*′	dimensionless coefficient [-]
*γ*	specific gravity of glass [kN/m^3^]
*μ*	Poisson’s ratio [-]
π	number “pi”
∑Δ*v*	change in gap volume [m^3^]
*σ*	stress [MPa]
*φ*	slope to horizontal [deg]
Subscripts and Markings	
0	initial gas parameters
1, 2 …	specific gas-filled gap
1-2, 2-3 …	glass pane (between gaps)
a	atmospheric
air	regarding air
ex	exterior glass pane
g	index that relates to glazing
in	interior glass pane
*n*	number of gas gaps
op	operating gas parameters
r	regarding circular IGU
v	index that relates to volume
w	index that relates to deflection
z	outside
γ	index that relates to specific gravity
